# Conjugation with Tris Decreases the Risk of Ketoprofen-Induced Mucosal Damage and Reduces Inflammation-Associated Methane Production in a Rat Model of Colitis

**DOI:** 10.3390/pharmaceutics15092329

**Published:** 2023-09-16

**Authors:** Melinda Ugocsai, Anett Bársony, Réka Anna Varga, Ámos Gajda, Noémi Vida, Norbert Lajkó, Benedek Rónaszéki, Gábor Tóth, Mihály Boros, Dániel Érces, Gabriella Varga

**Affiliations:** 1 Department of Orthopaedics, Albert Szent-Györgyi Medical School, University of Szeged, H-6725 Szeged, Hungary; 2 Department of Surgery, Albert Szent-Györgyi Medical School, University of Szeged, H-6725 Szeged, Hungary; 3 Institute of Surgical Research, Albert Szent-Györgyi Medical School, University of Szeged, H-6720 Szeged, Hungaryerces.daniel@med.u-szeged.hu (D.É.); 4 Second Department of Internal Medicine and Cardiology Center, Albert Szent-Györgyi Medical School, University of Szeged, H-6725 Szeged, Hungary; 5 Department of Medical Chemistry, Albert Szent-Györgyi Medical School, University of Szeged, H-6720 Szeged, Hungary; 6 ELKH-SZTE Biomimetic Systems Research Group, Albert Szent-Györgyi Medical School, University of Szeged, H-6720 Szeged, Hungary

**Keywords:** non-steroid anti-inflammatory drugs, mucosa, microcirculation, inflammation, TNBS colitis, methane generation, rat

## Abstract

We have designed a new compound from the non-steroidal anti-inflammatory drug (NSAID) ketoprofen (Ket) and 2-amino-2-(hydroxymethyl)-1,3-propanediol (Tris) precursors, with the aim to reduce the gastrointestinal (GI) side effects of NSAID therapies. We investigated mucosal reactions in a standard rat model of colitis together with methane generation as a possible indicator of pro-inflammatory activation under this condition (approval number: V./148/2013). Whole-body methane production (photoacoustic spectroscopy) and serosal microcirculation (intravital videomicroscopy) were measured, and mucosal damage was assessed (conventional histology; in vivo laser-scanning endomicroscopy). Inflammatory markers were measured from tissue and blood samples. Colitis induced an inflammatory response, morphological colonic damage and increased methane output. Ket treatment lowered inflammatory activation and colonic mucosal injury, but macroscopic gastric bleeding and increased methane output were present. Ket-Tris reduced inflammatory activation, methane emission and colonic mucosal damage, without inducing gastric injury. Conjugation with Tris reduces the GI side effects of Ket and still decreases the inflammatory response in experimental colitis. Methane output correlates with the mucosal inflammatory response and non-invasively demonstrates the effects of anti-inflammatory treatments.

## 1. Introduction

Non-steroidal anti-inflammatory drugs (NSAIDs) are effective antipyretic, analgesic and anti-inflammatory agents, but gastroduodenal complications, including erosions, ulcers and perforation, limit or prevent their everyday use [1,2]. In addition, upper gastrointestinal (GI) bleeding associated with the use of NSAIDs remains a serious clinical challenge with considerable mortality [3]. These potentially severe adverse events have prompted the development of a number of approaches to influence and reduce the extent of NSAID-linked mucosal damage. In this respect, previous animal studies have demonstrated that acetylsalicylic acid (ASA), chemically combined with tris-hydroxymethyl-aminomethane (Tris), has a less damaging effect on gastric mucosa than the original ASA with upkept anti-inflammatory bioactivity [4]. Furthermore, ASA-Tris conjugation has provided significant protection against the progression of cytokine-mediated inflammatory events in the large intestine, as well [5]. Ultimately, these results suggest that this method is suitable to decrease the GI side effects of ASA and that the procedure can be extended to other components of this class of drugs [6].

Ketoprofen (2-(3-benzoylphenyl) propanoic acid (Ket) is a frequently used NSAID with more potent anti-inflammatory activity than ASA [7]. Nevertheless, it is also among the most GI-toxic members of this class of drugs [8,9,10]. Therefore, our goal was to synthesize a new drug from Ket and Tris precursors according to the previously described protocol [4], with the aim of characterising and comparing the outcomes with those observed after the use of the original NSAID. In this context, we examined the effects of the Ket-Tris conjugate on functional and structural changes in the GI mucosa with a standardised baseline under in vivo conditions. Furthermore, we compared the efficacy of Ket-Tris administration versus equimolar doses of Ket in a rat model of experimental colitis to evaluate the therapeutic effects on pro-inflammatory mucosal changes in various segments of the GI tract.

An additional aim was to investigate a mechanism linked to the expected biological efficacy of Ket-Tris treatments. Here, we considered previous findings that methane may be liberated under pro-inflammatory circumstances or if there is a transient deficiency of oxygen delivery to the tissues [11,12]. Biogenic methane in the GI tract of mammals is produced by methanogenic archaea under strict anaerobic conditions [13,14]; nevertheless, well-documented studies with plant, animal and human cells have recently demonstrated that methane formation can also be a consequence of redox conditions accompanied by reactive oxygen species (ROS) formation, even in the presence of oxygen and without specific enzymes [11]. When released, GI methane is transferred through the splanchnic microcirculation to the lungs and partially exhaled if the partial pressure is higher than that in the atmosphere, together with other amounts discharged from the skin and body orifices [15]. Since local ROS formation is among the first homeostatic responses during GI microcirculatory distress and inflammation, we hypothesised that the monitoring of methane formation might also be an indicator of ROS-driven mucosal processes. Hence, we carried out a sequential exploration of the in vivo dynamics of methane generation, together with pro-inflammatory mucosal changes in experimental colitis, and studied the effects of Ket and Ket-Tris administration in this scenario.

## 2. Materials and Methods

### 2.1. Chemical Synthesis and Characterisations of Ket-Tris Conjugate

**Figure 1:**
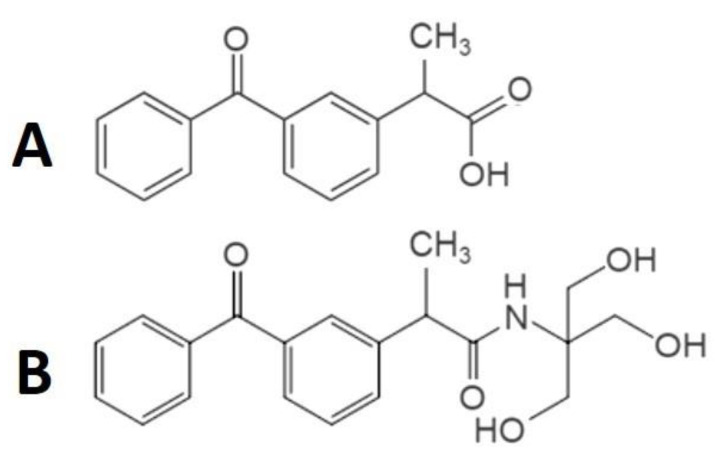
Structural formulae. Structural formulae for the Ket (**A**) and developed Ket-Tris (**B**) conjugate.

The synthesis and the supporting NMR and HPLC studies were carried out at the Department of Medical Chemistry, University of Szeged. The procedures are described in detail in a previous paper [4]. Briefly, 5.09 g ketoprofen (*RS*)-2-(3-benzoyl phenyl)propanoic acid was dissolved in 200 mL abs THF and cooled to −15 °C. Under stirring, 2.62 mL isobutylchloroformate and 2.79 mL trimethylamine were added. After 25 min stirring at -15 °C, 2.42 g of tris hydroxymethyl amino methane was added and was stirred for an additional one hour at 0 °C. The stirring was continued at RT overnight. The reaction mixture was filtered and evaporated, and the resulting crystalline material was washed with diethyl ether-hexane, resulting in the pure product: 2.9 g, 40.56%, Ms 358.06 (Figure 1).

^1^H NMR (600 MHz, DMSO): δ = 1.34 (d, *J* = 7.01 Hz, 3H, CH–CH_3_), 3.50 (dd, *J* = 5.87, 10.91 Hz, 3H, CH_2_–OH), 3.55 (dd, *J* = 5.87, 10.91 Hz, 3H, CH_2_–OH), 3.88 (q, *J* = 7.04 Hz, 1H, CH–CH*_3_*), 4.71 (t, *J* = 5.66 Hz, 3H, CH_2_–OH), 7.36 (s, 1H, NH), 7.49–7.76 (m, 9H, H–2, H–4, H–5, H–6, Ar); ^13^C NMR (150 MHz, DMSO): δ = 19.3 (CH–CH_3_), 45.3 (CH_2_), 61.0 (CH_2_–OH), 62.6 (C–CH_2_–OH), 126.5 (C–5), 129.0 (Ar–C–2, Ar–C–4, Ar–C–6), 130.2 (C–6, Ar–C–3, Ar–C–5), 132.2 (C–2), 133.2 (C–4), 137.3 (C–3), 137.5 (Ar), 143.2 (C–1), 174.8 (C=O–NH), 196.2 (C=O) (for NMR spectra, see Figure S1 in Supplementary Materials).

HPLC: solvents: A: 0.1% TFA in water, B: 0.1% TFA, 80% AcN in water; column: Phenomenex Luna 10 µ C 18 100 Å; gradient 20–100% B in 20 min, flow 1.2 mL/min, detection at 220 nm R_t_ 10.38 min.

### 2.2. In Vivo Studies—Animals

**Figure 2:**
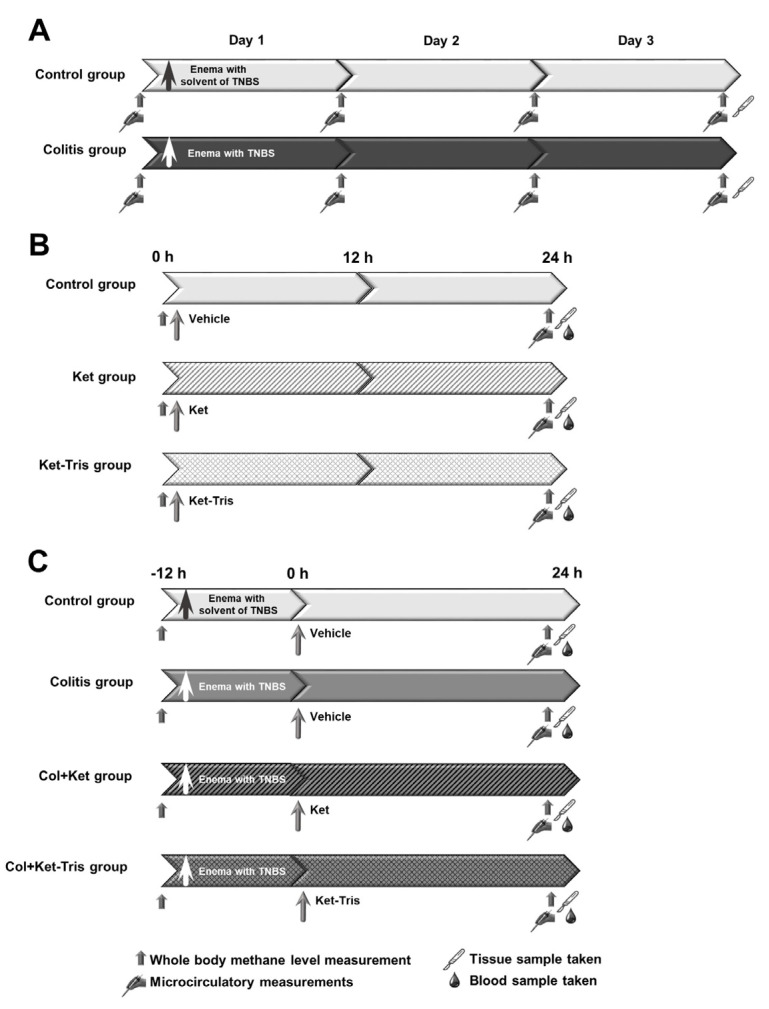
Experimental protocols. Experimental protocol for Study 1 (**A**), Study 2 (**B**) and Study 3 (**C**).

The experiments were performed in three separate, but interlinked, studies (Figure 2A–C) on male Sprague–Dawley rats (average weight 200 g ± 10 g; n = 82), housed in transparent plastic cages under a 12 h dark–light cycle in a thermoneutral environment (21 ± 2 °C). The animals were kept on normal laboratory chow, and then all of them were fed with a carbohydrate-rich diet (bread rolls) for three days prior to the experiments. In the case of anaesthesia, the animals were deprived of food, but not water, for 12 h prior to the experiments. The protocols conformed with EU directive 2010/63 for the protection of animals used for scientific purposes and were approved by the National Scientific Ethical Committee on Animal Experimentation (National Competent Authority) under licence number V./148/2013 (date of approval: 8 January 2013). This study also complied with the US National Institutes of Health Guidelines for the Care and Use of Laboratory Animals criteria. In the present study, the updated ARRIVE 2.0 guidelines were followed (see File S1 in Supplementary Materials). The sample sizes were estimated with PS: Power and Sample Size Calculation 3.1 software [16], and, for sample size estimation, the differences in the tissue MPO activity were the primary outcome measures. A random number generator (Randomiser.org) was used to allocate the animals to the respective groups. Until the start of the treatments, only one experimenter (GV) knew which group the animals belonged to. No animals were excluded (exclusion criterion: wellbeing score over 7; File S2 in Supplementary Materials) or lost during the pilot studies and the experiments.

### 2.3. Experimental Protocol 1

In Study 1, the animals (n = 40) were randomly allocated into two groups (n = 20 each). In group 1, colitis was induced with the intracolonic (ic) administration of 2-,4-,6-trinitrobenzene-sulfonic acid (TNBS, 40 mg/kg in 0.25 mL of 25% ethanol) through an 8 cm long, soft, plastic catheter under transient light inhalation anaesthesia (Morris et al. 1989). In sham-operated group 2, the animals received enemas with a total volume of 0.25 mL containing 25% ethanol (the solvent for TNBS). The animals were then returned to their cages and fed standard laboratory chow ad libitum. On days 1, 2 and 3 after colitis induction, five animals from each group were anaesthetised (sodium pentobarbital; 50 mg/kg i.p.) for invasive microcirculatory investigations and biochemical sample collection. Further, whole-body methane detection was performed individually in the same timeframes (see Section 2.8) (Figure 2A).

### 2.4. Experimental Protocol 2

In Study 2, the animals (n = 18) were randomly allocated into three groups (n = 6 each). Group 1 served as a vehicle-treated control, where 10 mL/kg of buffered 0.11 M potassium hydroxide (KOH) was administered orally. In group 2, high doses of Ket solution (0.08 mmol/kg, in a volume of 10 mL/kg) were gavaged via a flexible oesophageal tube to the animals. After the treatment, the animals were returned to their cages and fed a carbohydrate-rich diet ad libitum. Group 3 was treated with the Ket-Tris conjugate in equimolar doses to Ket (0.09 mmol/kg, in a volume of 10 mL/kg). The pH of both the Ket and Ket-Tris solutions was set to 7.4 with KOH. Whole-body methane generation was detected at the beginning of the observations and before and after the treatments on days 1 and 2. On day 2, after methane output measurements, the animals were anaesthetised with sodium pentobarbital (50 mg/kg i.p.). For instrumentation, the animals were placed in a supine position on heating pads, and the trachea and right jugular vein were cannulated to secure spontaneous breathing and IV administration of fluids and fluorescence dye, respectively. After a midline abdominal incision, intravital videomicroscopy was performed to examine the microcirculatory changes on the serosal surfaces of the stomach, duodenum, jejunum, ileum and colon. The stomach was opened along the great curvature and rinsed with saline to remove the gastric contents, and in vivo histology of the gastric mucosa was performed in each group by fluorescent confocal laser scanning endomicroscopy (CLSEM). At the end of the protocol, tissue biopsies were obtained from the stomach, and blood samples (0.5 mL) were taken from the inferior vena cava for further investigation (Figure 2B).

### 2.5. Experimental Protocol 3

In Study 3 (n = 24), the control group (n = 6) received enemas with a total volume of 0.25 mL containing 25% ethanol (the solvent for TNBS). In groups 2, 3 and 4 (n = 6 each), colitis was induced with a TNBS enema (40 mg/kg). In groups 3 and 4, the animals were gavaged with Ket (Col + Ket; 0.08 mmol/kg, 20 mg/kg, in a volume of 10 mL/kg) or Ket-Tris conjugate (Col + Ket-Tris; 0.09 mmol/kg, 30 mg/kg, in a volume of 10 mL/kg) 12 h after colitis induction. The animals in the control and non-treated colitis groups were gavaged with the solvent for Ket (10 mL/kg buffered 0.11 M of potassium hydroxide). On day 2 (24 h after Ket or Ket-Tris treatments), the animals were anaesthetised (sodium pentobarbital; 50 mg/kg i.p.), and surgery was performed. They were placed in a supine position on heating pads, the trachea and the right jugular vein were cannulated, and the lumen of the distal colon was exposed after a midline abdominal incision. Then, the mucosa was rinsed with saline to remove the bowel contents. In each group, in vivo histology of the colonic mucosa was performed (see Section 2.7) to examine the changes in the microvasculature and superficial morphology. At the end of the experiments, full-thickness tissue samples were taken to measure mucosal biochemical changes, and venous blood samples were taken to determine the plasma TNF-α level (Figure 2C).

### 2.6. Direct Measurements on the GI Microcirculation

In Studies 1 and 2, the orthogonal polarisation spectral (OPS) imaging technique (Cytoscan A/R, Cytometrics, Philadelphia, PA, USA) was used for non-invasive visualisation of the serosal microcirculation of the stomach, duodenum, jejunum, ileum or colon. This technique utilises reflected polarised light at the wavelength of the isobestic point of oxy- and deoxyhaemoglobin (548 nm). As polarisation is preserved in reflection, only photons, scattered from a depth of 2–300 mm, contribute to image formation. A 10× objective was placed onto the serosal surface of the GI organs, and microscopic images were recorded with an S-VHS video recorder 1 (Panasonic AG-TL 700; Matsushita Electric Ind. Co., Ltd., Osaka, Japan). Quantitative assessment of the microcirculatory parameters was performed off-line by frame-to-frame analysis of the videotaped images. Red blood cell velocity (RBCV; μm/s) changes in the postcapillary venules were determined in three separate fields by means of a computer-assisted image analysis system (IVM Pictron, Budapest, Hungary) [4].

In Study 3, microcirculatory videos were recorded with an incident dark-field (IDF)-imaging device (Cytocam, Braedius medical, Huizen, The Netherlands), in accordance with the international recommendations [17]. The video files were saved directly to the Braedius CytoCam HDD in AVI format. Optical magnification of 4x was used to provide a 1.55 × 1.16 mm field of view. The MFI is a semi-quantitative and summarised score, which is the mean value of four quadrant measurements. Categorical values are provided based on predominant flow (no flow = 0, intermittent = 1, sluggish = 2, continuous = 3). De Backer’s score (in 1/mm) was calculated as the number of vessels crossing three arbitrary horizontal and three vertical equidistant lines (drawn on the screen) divided by the total length of the lines.

### 2.7. In Vivo Detection of Mucosal Damage

The extent of damage of the gastric mucosa was evaluated by means of fluorescence CLSEM (Optiscan Five1, Optiscan Pty. Ltd., Melbourne, Victoria, Australia) developed for in vivo histology. The analysis was performed twice, separately by two investigators (GV and MU). The mucosal surface of the stomach was surgically exposed and laid flat for examination. The microvascular structure was recorded after IV administration of 0.3 mL of fluorescein isothiocyanate-dextran (FITC-dextran, 150 KDa, 20 mg/mL solution dissolved in saline; Sigma-Aldrich Chemie, Schnelldorf, Germany). The objective of the device was placed onto the mucosal surface of the stomach, and confocal imaging was performed 5 min after dye administration (1 scan/image, 1024 × 512 pixels and 475 × 475 μm per image). The changes in the mucosal architecture were examined following topical application of the fluorescent dye acridine orange (Sigma-Aldrich Inc., St. Louis, MO, USA). The surplus dye was washed off the mucosal surface of the stomach with saline 2 min before imaging. Non-overlapping fields’ gastric and colon mucosa were processed in Ket-treated animals and compared with the samples of the control or Ket-Tris conjugate-treated group using a semiquantitative scoring system, as described previously [18]. We employed three criteria: (1) the structure of the microvessels (0 = normal; 1 = dye extravasation, but the vessel structure is recognisable; 2 = destruction, and the vessel structure is unrecognisable); (2) oedema (0 = no oedema, 1 = moderate epithelial swelling, 2 = severe oedema); and (3) epithelial cell outlines (0 = normal, clearly well-defined outlines; 1 = blurred outlines; 2 = lack of normal cellular contours).

### 2.8. Whole-Body Methane Emissions Measurement

We employed a purpose-built, near-infrared laser technique-based PAS (photoacoustic spectroscopy) apparatus [19,20]. PAS is a subclass of optical absorption spectroscopy that measures optical absorption indirectly via the conversion of absorbed light energy into acoustic waves due to the thermal expansion of absorbing gas samples. The amplitude of the generated sound is directly proportional to the concentration of the absorbing gas component. The gas sample passes through the photoacoustic cell via a stainless steel tube. Gas samples were taken from the sampling chamber with an internal volume of 2510 cm^3^. Before placing the animals into the chamber, the methane concentration of the gas in the chamber (room air) was determined and applied as the value of the background methane concentration and was subtracted from the value of the methane emissions of the animals. Then, the rat was placed in the chamber, it was sealed, and a sample of the chamber gas was analysed exactly 5 min later (a period of 5 min was sufficient for reliable and reproducible measurements in pilot studies). The rat was then removed, and the chamber was thoroughly ventilated with room air for 2 min between animals [19].

### 2.9. In Vitro Studies

#### 2.9.1. Tissue Biopsies

Stomach and colon biopsies were kept on ice, then homogenised in phosphate buffer (pH 7.4), which contained 50 mM Tris-HCl (Reanal, Budapest, Hungary), 0.1 mM EDTA, 0.5 mM dithiotreitol, 1 mM phenylmethylsulfonyl fluoride, 10 μg/mL soybean trypsin inhibitor and 10 μg/mL leupeptin (Sigma-Aldrich GmbH, Germany). The homogenate was centrifuged at 4 °C for 20 min at 24,000× *g*, and the supernatant was loaded into centrifugal concentrator tubes (Amicon Centricon-100; 100,000 MW cut-off ultrafilter, Millipore, Burlington, MA, USA).

#### 2.9.2. Tissue Xanthine Oxidoreductase (XOR) Activity

Stomach and colon biopsies were homogenised in phosphate buffer (pH 7.4) containing 50 mM Tris-HCl, 0.1 mM EDTA, 0.5 mM dithiotreitol, 1 mM phenylmethylsulfonyl fluoride, 10 μg ml-1 soybean trypsin inhibitor and 10 μg ml-1 leupeptin. The homogenate was centrifuged at 4 °C for 20 min at 24,000× *g*, and the supernatant was loaded into centrifugal concentrator tubes. XOR activity was determined in the ultrafiltered supernatant by a fluorometric kinetic assay, based on the conversion of pterine to isoxanthopterine in the presence (total XOR) or absence (XO activity) of the electron acceptor methylene blue [21].

#### 2.9.3. Tissue Myeloperoxidase (MPO) Activity

The myeloperoxidase (MPO) activity was measured in gastric and colon biopsies with the method developed by Kuebler et al. [22]. Briefly, the tissue was homogenised with Tris-HCl buffer (0.1 M, pH 7.4) containing 0.1 M polymethylsulfonyl fluoride to block tissue proteases and then centrifuged at 4 °C for 20 min at 24,000× *g*. The MPO activities of the samples were measured at 450 nm (UV-1601 spectrophotometer; Shimadzu, Japan), and the data were referred to the protein content.

#### 2.9.4. Tissue Nitrite/Nitrate (NO_x_) Levels

Nitrite and nitrate (NO_x_) stable end products of NO were determined in gastric and colon homogenate by the Griess reaction. This assay depends on the enzymatic reduction of nitrate to nitrite, which is then converted into a coloured azo compound that is detected spectrophotometrically at 540 nm. Total NO_x_ was calculated and expressed as µmol/mg protein [23].

#### 2.9.5. Tissue Cytochrome *c* Oxidase (Cyt*c*) Level

The release of Cyt*c* was calculated via the time-dependent oxidation of Cyt*c* at 550 nm, as described previously [24]. Gastric and colon tissue samples were homogenised in 10× ice-cold MitOx2 medium with a Potter grinder and then centrifuged at 800 g for 5 min at 4 °C. Then, 50 μL supernatant was added to 2.5 mL Cyt*c* stock solution (10.6 mg Cyt*c* dissolved in 20 mL distilled water) (Sigma-Aldrich, Budapest, Hungary), and the decrease was measured spectrophotometrically in the optical density at 550 nm during 1 min intervals at 0, 30 and 60 min.

#### 2.9.6. Tissue Malondialdehyde (MDA) Assay

MDA production is associated with oxidative damage of lipid membranes and, thereby, the degree of lipid peroxidation. The MDA level was measured through the reaction with thiobarbituric acid (TBA), and the values were corrected for the tissue protein content. The MDA concentration was determined on a standard curve (nmol/mL) [25].

#### 2.9.7. Plasma Tumour Necrosis Factor Alpha (TNF-α) Level

Blood samples (0.5 mL) were taken from the inferior caval vein into precooled, heparinised (100 U/mL) polypropylene tubes, then were centrifuged at 1000× *g* at 4 °C for 30 min and stored at −70 °C until assay. Plasma TNF-α concentrations were determined in duplicate by means of a commercially available, enzyme-linked, immunosorbent assay (Quantikine ultrasensitive ELISA kit for rat TNF-α; Biomedica Hungaria Kft, Budapest, Hungary), according to the manufacturer’s instructions. The minimum detectable level was less than 5 pg/mL, and the interassay and intraassay coefficients of variation were less than 10%.

### 2.10. Statistical Analysis

A statistical software package (SigmaStat for Windows 3.5, Jandel Scientific, Erkrath, Germany) was used for data analysis. Within the groups, the Friedman repeated measures analysis of variance on ranks was applied. Time-dependent differences from the baseline for each group were assessed by Dunn’s method. Differences between groups were analysed with the Kruskal–Wallis one-way analysis of variance on ranks, followed by Dunn’s method for pairwise multiple comparison. In the figures, median values and 75th and 25th percentiles are given; *p* values < 0.05 were considered significant.

## 3. Results

### 3.1. Study 1

#### 3.1.1. Changes in Inflammatory Mediators after Colitis Induction

**Figure 3:**
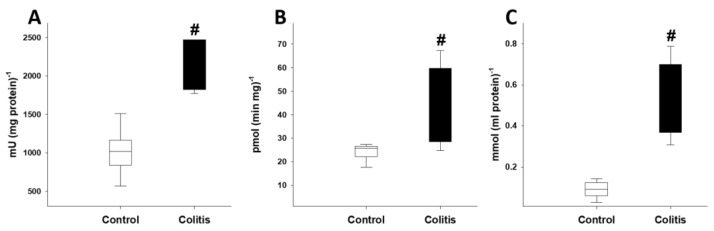
Colonic biochemical changes in Study 1. MPO activity (**A**), XOR activity (**B**) and MDA level (**C**) in the control (empty box) and colitis (black box) groups. The plots demonstrate the median (horizontal line in the box) and the 25th (lower whisker) and 75th (upper whisker) percentiles. # *p* < 0.05 between the colitis group vs the control group.

On day 3, after colitis induction, significant elevation was demonstrated in the level of MPO and XOR activity and MDA levels relative to the control group values (Figure 3A–C).

#### 3.1.2. Changes in Microcirculatory Parameters

**Figure 4:**
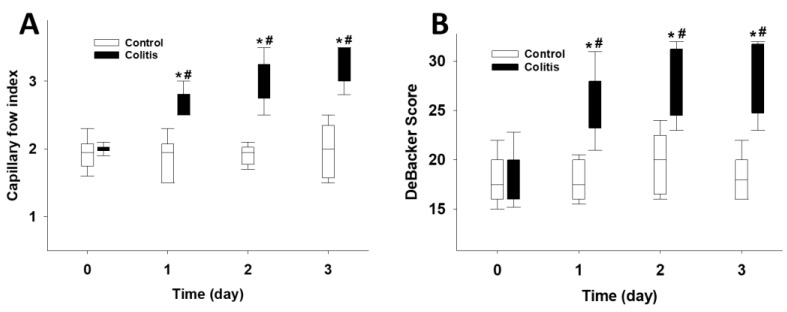
Changes in microcirculatory parameters in Study 1. Changes in capillary flow index (**A**) and DeBacker score (**B**) in the control (empty box) and colitis (black box) groups. The plots demonstrate the median (horizontal line in the box) and the 25th (lower whisker) and 75th (upper whisker) percentiles. * *p* < 0.05 for groups vs. baseline values. # *p* < 0.05 between the colitis group vs. the control group.

A significant increase was observed in the capillary flow index and DeBacker’s score values in the colitis group on days 1, 2 and 3 of colitis relative to the control values (Figure 4A,B).

#### 3.1.3. Changes in Whole-Body Methane Emission

**Figure 5:**
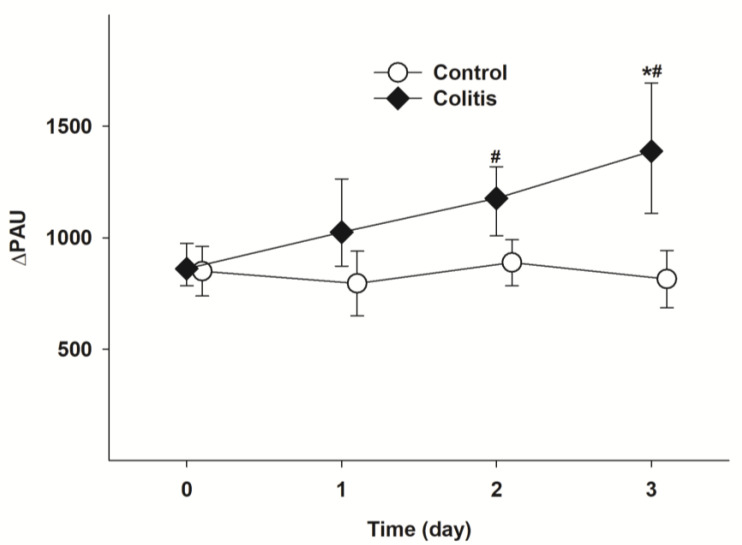
Changes in whole-body methane emissions. Changes in whole-body methane emissions in the control (white circle with solid line) and colitis (black diamond with solid line) groups. The plots demonstrate the median (horizontal line in the box) and the 25th (lower whisker) and 75th (upper whisker) percentiles. * *p* < 0.05 for groups vs. baseline values. # *p* < 0.05 between the colitis group vs. the control group.

Similarly, whole-body methane emissions were significantly elevated on days 2 and 3 after colitis induction, in contrast to the baseline values and values of the control group (Figure 5).

### 3.2. Study 2—Effects of Ket-Tris Conjugate

#### 3.2.1. In Vivo Detection of Gastric Mucosal Injury

**Figure 6:**
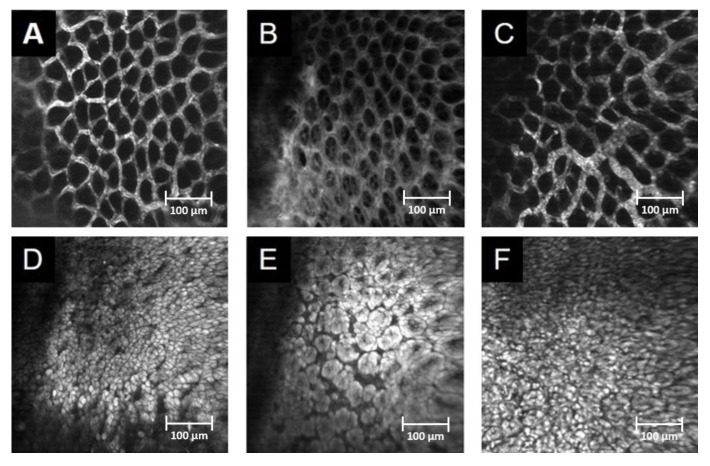
In vivo detection of gastric mucosal injury in Study 2. In vivo histology images of the mucosal surface of the stomach recorded by confocal laser scanning endomicroscopy (CLSEM) after IV administration of FITC-dextran (**A**–**C**) or topical administration of acriflavine (**D**–**F**). (**A**,**D**) The normal mucosal vasculature and normal structure of the mucosa in the control group. (**B**,**E**) Dye leakage from the vessel lumina, loss of epithelium and oedema formation on the surface after Ket treatment. (**C**,**F**) Normal structure of the mucosal vasculature and normal mucosa surface after Ket-Tris treatment.

The morphological changes in the gastric mucosa were evaluated by means of in vivo imaging, using CLSEM. The FITC dextran and acriflavine staining demonstrated significant tissue damage in the Ket group. A severely injured capillary network, fluorescent dye leakage, elevated endothelial permeability and oedema formation (Figure 6B,E) were observed, in contrast to the normal mucosal pattern of the control group (Figure 6A,D). The Ket-Tris treatment effectively attenuated the structural damage or morphological changes in gastric mucosa (Figure 6C,F, Table 1).

#### 3.2.2. Changes in Biochemical Parameters

Pro-inflammatory biochemical markers, MPO, XOR, NO_x_, MDA, Cyt*c* and TNF-α 2 were significantly increased 24 h after Ket treatment relative to the control group. Ket-Tris administration did not induce elevation of these molecules (Table 1).

#### 3.2.3. In Vivo Detection of the Microcirculation

RBCV of the serosa was measured as a quantitative marker of the GI microcirculatory condition. The RBCV in gastric serosa was significantly raised in the Ket-treated group as compared to the control group (Table 2), and a similar change was detected in the duodenum, jejunum and ileum, but not in the colon. Baseline microcirculatory values were measured in the entire GI tract in the Ket-Tris-treated group (Table 2).

#### 3.2.4. Changes in Whole-Body Methane Emission

**Figure 7:**
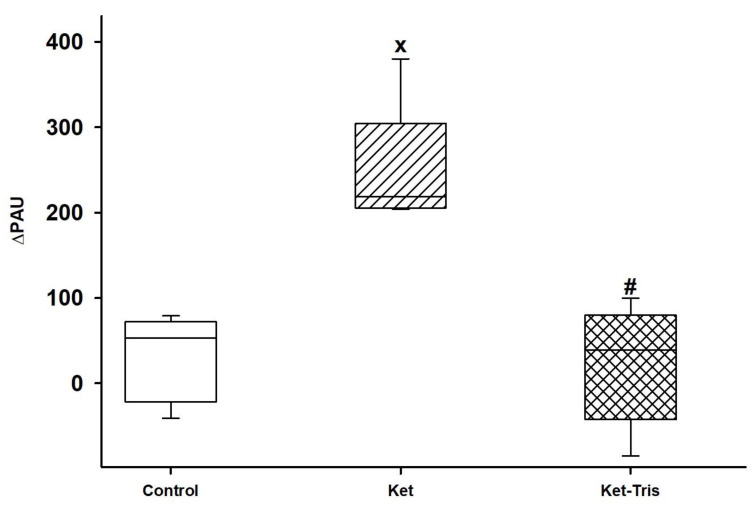
Changes in whole-body methane emission in Study 2. Changes in whole-body methane emission: the difference between two successive measurement times is shown in the control (empty box), Ket-treated (striped box) and Ket-Tris-treated (checked box) groups. The plots demonstrate the median (horizontal line in the box) and the 25th (lower whisker) and 75th (upper whisker) percentiles. x *p* < 0.05 between groups vs. the control group, # *p* < 0.05 between the Ket-treated group vs. the Ket-Tris-treated group.

The whole-body methane level was measured before and 24 h after the treatments. In the control and Ket-Tris groups, no changes were detected at 24 h, while significant elevation was demonstrated 24 h after treatment in the Ket-treated group (Figure 7).

### 3.3. Study 3

#### 3.3.1. Changes in Biochemical Factors

**Figure 8:**
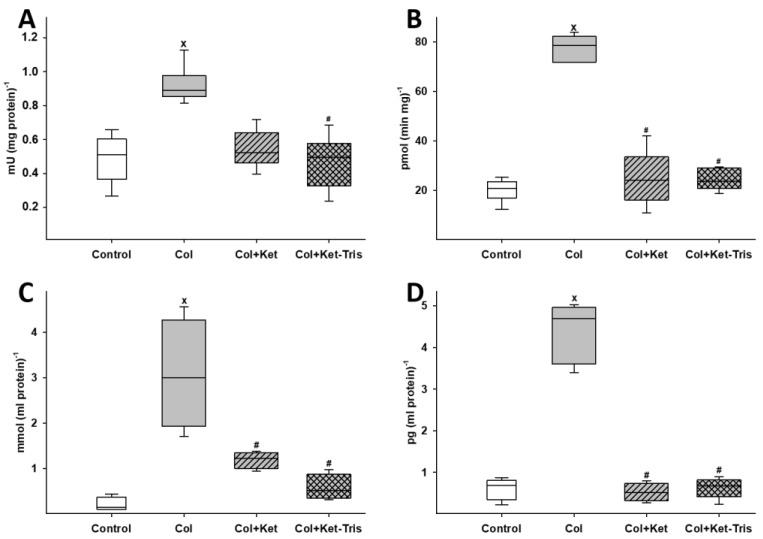
Changes in biochemical factors in Study 3. Changes in colonic MPO activity (**A**) and XOR activity (**B**), levels of MDA (**C**) and Cyt*c* (**D**) in the control (empty box), colitis (grey empty box), Ket-treated colitis (striped grey box) and Ket-Tris-treated colitis (checked grey box) groups. The plots demonstrate the median (horizontal line in the box) and the 25th (lower whisker) and 75th (upper whisker) percentiles. x *p* < 0.05 between groups vs. the control group, # *p* < 0.05 between the non-treated colitis group vs. the Ket- or Ket-Tris-treated group.

Twenty-four hours after colitis induction, significant elevation was demonstrated in the level of MPO and XOR enzyme activity, MDA level and Cyt*c* level relative to the control group values (Figure 8A–D). In the Ket-treated colitis group, significantly decreased XOR enzyme activity, MDA and Cyt*c* levels were observed. The Ket-Tris treatment effectively reduced all of the observed parameters.

#### 3.3.2. In Vivo Detection of Mucosal Damage in the Colon and Serosal Microcirculation with In Vivo Microscopy

**Figure 9:**
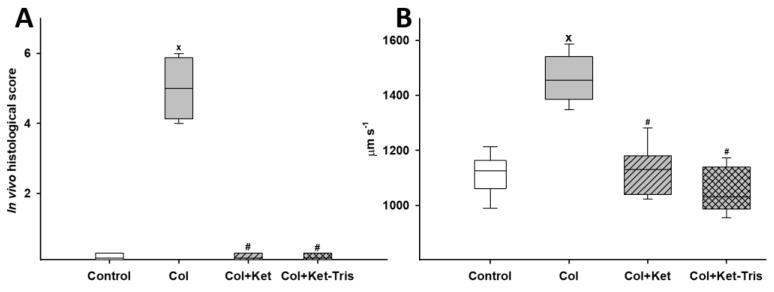
Changes in mucosal in vivo histological score in Study 3. Changes in mucosal in vivo histological score (**A**) and serosal red blood cell velocity (**B**) in the control (empty box), colitis (grey empty box), Ket-treated colitis (striped grey box) and Ket-Tris-treated colitis (checked grey box) groups. The plots demonstrate the median (horizontal line in the box) and the 25th (lower whisker) and 75th (upper whisker) percentiles. x *p* < 0.05 between groups vs. the control group, # *p* < 0.05 between the non-treated colitis group vs. the Ket- or Ket-Tris-treated group.

After TNBS enemas, severe mucosal injury was present 24 h after TNBS induction. Fluorescent dye leakage with oedema formation and the complete loss of the epithelium were commonly observed. In the control (vehicle-treated) group, the normal structure of capillaries and the crypts covered by epithelial cells were visualised. Ket and Ket-Tris treatments prevented structural changes in the microvasculature and the loss of epithelium of the inflamed colonic mucosa. Figure 9A shows the score for the in vivo histological examination. The RBCV in the colonic subserosa was significantly raised in the colitis group as compared to the control group. Ket and Ket-Tris treatment decreased the elevated RBCV by the end of the observation period (Figure 9B).

#### 3.3.3. Changes in Whole-Body Methane Emissions

**Figure 10:**
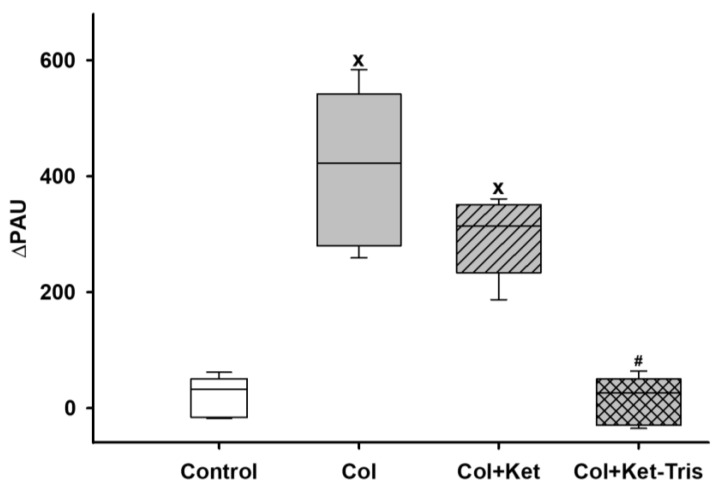
Changes in whole-body methane emissions in Study 3. Changes in whole-body methane emissions in the control (empty box), colitis (grey empty box), Ket-treated colitis (striped grey box) and Ket-Tris-treated colitis (checked grey box) groups. The plots demonstrate the median (horizontal line in the box) and the 25th (lower whisker) and 75th (upper whisker) percentiles. x *p* < 0.05 between groups vs. the control group, # *p* < 0.05 between the non-treated colitis group vs. the Ket- or Ket-Tris-treated group.

Significant whole-methane emissions were detected after 24 h of colitis induction in the colitis and Ket-treated colitis groups, in contrast to the control group. The Ket-Tris treatment reduced the level of methane output and did not result in a significant change in methane emissions (Figure 10).

## 4. Discussion

Our main goal was to boost the safety of NSAID use. Ket and its derivatives are commonly used worldwide; the good analgesic and antipyretic efficacies of the compounds have been well documented for a variety of acute and chronic pain conditions and are also the main components of anti-inflammatory treatments in many musculoskeletal diseases [26,27,28]. However, as a member of the NSAID family, adverse reactions, including GI side effects, are still apparently unavoidable [29]. To this end, we analysed the effects of Ket and its chemically modified Ket-Tris counterpart on GI mucosal changes in a cross-sectional study via in vivo tests in rodents. Ket gavage caused significant endothelial and epithelial injury in the rat stomach, and the matching microcirculatory dysfunction was accompanied by elevated MPO levels, indicating PMN accumulation in the gastric mucosa. The enhanced activity of XOR, a major ROS-producing enzyme, elevated tissue MDA and NO_x_ levels. Meanwhile, Cyt*c* release demonstrated the presence of nitroxidative stress, and the higher circulating plasma TNF-α concentrations showed the spread of local inflammatory signals.

It was found that Ket-Tris conjugate applied in the same way did not result in the development of such pro-inflammatory processes; did not increase MPO and XOR activity, MDA, Cyt*c* and NO_x_; and left plasma TNF-α levels unchanged. Further, RBCV was kept at the control level, and endothelial injury of GI mucosa was not observed. In conclusion, these data provided evidence that conjugation with Tris reduces the ulcerogenic characteristic of Ket; the newly developed compound has significantly lower mucosa-damaging potential as compared to the original NSAID.

To date, a large body of literature has addressed the strategies to reduce the risk of GI side effects of NSAIDs, such as increasing their bioavailability [30] and combined therapy with other analgesics or adjuvant agents [31], but there is still a need for effective chemical modifications with inherently lowered ulcerogenic properties. Current ulcer-prevention management is usually based on “modified release” enteric-coating formulations or additional acid secretion inhibitors and antacids, but a proper multimodal therapy is often challenging in non-compliant patients [32,33]. Molecular modifications offer another possibility because it is accepted that the acidic characteristic of the carboxyl group is a major ulcerogenic factor of this class of drugs. Indeed, several modified compounds were examined in experimental settings in recent decades, and some of them (such as the 1,3,4-oxadiazole derivate of diclofenac) have shown significantly reduced ulcerogenic effects [34,35]. Our approach with chemical conjugation of the amino alcohol Tris with Ket falls into this category. Tris has well-known effects on arterial pH and the base deficit in mixed acidosis and may also inhibit enzyme activities, such as aminopeptidases and alpha-amylases. More importantly, earlier studies from our laboratory have demonstrated that a combination of Tris with acetylsalicylic acid (ASA) exhibits strong bioactivity against cytokine-mediated inflammatory events, with less harmful consequences for the GI mucosa [4]. In another report, Tris-ASA conjugation has been shown to mitigate ASA-induced oxidative stress and perturbed mitochondrial respiratory activity [5]. Using this background as a basis, we hypothesised that this methodology might also provide a therapeutic benefit in other NSAIDS. The results demonstrated a significant reduction in drug-induced mucosal damage and complication rates when the new molecule was used.

Another, novel aspect of this study is the real-time detection of whole-body methane emissions in a sufficiently large cohort of experimental animals. It is widely accepted that the bulk of the methane produced in the mammalian intestines is excreted via the lungs. Breath testing has, therefore, become a tool for the diagnosis of certain GI conditions. Nevertheless, as a consequence of its physicochemical properties, methane is distributed evenly across membrane barriers and traverses the mucosa freely; thus, the production of it is reflected not only in the exhaled air, but also in its passage through body surfaces [36]. Furthermore, a correlation between the carrier capacity of GI microcirculation and the concentration of methane in the exhaled air has recently been described [37]. Our analytical approach with specific PAS detection reflected both the dynamics of production and the overall profile of net methane generation in various groups of unrestrained animals [19]. In this set-up, significantly higher whole-body methane output was identified after oral Ket treatments, while this change was not observed for Ket-Tris administration.

Traditionally, GI methane formation has been regarded as the enzymatic product of the metabolism of strictly anaerobic methanogenic archaea, a unique class of prokaryotes [13,14]. However, a wealth of data accumulated during the past decade has unambiguously confirmed direct methane release from eukaryotes in the absence of microbes and in the presence of oxygen [11]. More recently, conclusive evidence was provided for biotic methane formation across all multicellular lifeforms, even under aerobic conditions [38,39]. Methane has previously been shown to form in chemical model systems when methyl group-containing compounds are incubated with ferric iron, ascorbate and hydrogen peroxide in the absence of enzymes [40]. Ernst et al. have demonstrated that ROS-induced methyl radicals, which are derived from organic compounds containing sulphur- or nitrogen-bonded methyl groups, are key intermediates that ultimately lead to methane production in all eukaryotes, including human cell lines [41,42]. The biological significance of the reaction is still not fully understood, but the totality of data now suggests that methane excretion in the breath of mammals reflects intestinal bacterial fermentation, plus an unknown and variable amount of de novo generation induced from metabolically active target cells in conditions associated with oxidative stress [19,42]. This interpretation indicates that the signal intensities of Ket-Tris-linked pro-inflammatory responses are significantly lower than those after the administration of the original Ket molecule.

Another goal of this protocol was to test the therapeutic potential of Ket-Tris in a health condition where the intestines are inflamed. To this end, TNBS-induced experimental colitis, a standardised inflammatory stress induction method, was used, where various degrees of mucosal damage are expected in different regions of the GI tract. In this protocol, a TNBS enema caused severe mucosal damage, with higher inflammatory enzyme activities in the colon. This process was accompanied by hyperaemic microcirculation in the serosa, influx of PMN leukocytes and the activation of ROS-generating XOR. In addition, whole-body methane emissions rose significantly as compared to the vehicle-treated control animals. Based on earlier findings [37] and these hallmark signs of inflammation, we suggest that the elevated methane output in this in vivo system is partly due to the inflammatory hyperaemia affecting the large bowel and partly due to local ROS generation within the bowel wall. This interpretation can also explain the increase in the Ket-treated colitis group and the decreased methane emissions after Ket-Tris administration in animals with colitis, as significantly stronger anti-inflammatory effects were demonstrated for Ket-Tris in Study 2. The influence of the Ket-Tris conjugate can, therefore, be more pronounced on the colonic microcirculation, hence resulting in a lower level of methane emissions. Thus, we propose that real-time monitoring of the exhaled methane level may offer appropriate means to examine and follow the ROS-driven mucosal inflammatory processes in the GI system and to monitor the effect of different therapies, at least in rodents.

The limitations of the study should also be discussed because only a relatively short follow-up period was used in this complex scenario, and further research is needed to identify the exact intracellular and molecular mechanism of mucosa defence/repair in association with the protective action of Tris conjugation. Additional investigations of gene signatures and transcriptional responses, relevant topical cytotoxic events involving the mucus–bicarbonate barrier, and cyclo-oxygenase (COX) pathway markers can be used to discover further important details after compound treatments.

## 5. Conclusions

In conclusion, the results reported in this paper confirm the specific effect of Tris in the protection of the GI tract from NSAID-induced damage. It is conceivable that Tris conjugation results in upkept cell viability and preserved mucosal morphology as compared to the more harmful effect of the original NSAID. In these experiments, the net methane production was detected, and the whole-body methane output was closely connected to the actual inflammatory status and the microcirculation of the GI mucosa. In the Ket-Tris-treated animals, the production of methane was kept at the level of the sham-operated group, which points to a role of methane as an alarm signal during the development of pro-inflammatory responses and a possible indication of the need for anti-inflammatory measures to influence such events.

## Figures and Tables

**Table 1:** Extent of tissue damage and changes in biochemical parameters in Study 2.

	Parameters	Control	Ket	Ket-Tris
**Tissue damage**	**Median** *25p*; *75p*	**0.15** *0*; *0.3*	**5 ^a^** *4.12*; *5.87*	**0.275 ^b^** *0*; *0.3*
**MPO**	**Median** *25p*; *75p*	**1.7** *1.06*; *1.85*	**2.4 ^a^** *2.16*; *2.16*	**1.6 ^b^** *1.5*; *1.83*
**XOR**	**Median** *25p*; *75p*	**18.7** *14.9*; *27.2*	**41.9 ^a^** *33.9*; *46.2*	**23.5 ^b^** *18.9*; *26.1*
**NO_x_**	**Median** *25p*; *75p*	**0.62** *0.51*; *20*	**1.37 ^a^** *1.14*; *1.84*	**0.65 ^b^** *0.61*; *0.68*
**MDA**	**Median** *25p*; *75p*	**0.21** *0.15*; *0.42*	**0.88 ^a^** *0.81*; *1.38*	**0.35 ^b^** *0.29*; *0.39*
**Cyt*c***	**Median** *25p*; *75p*	**0.68** *0.34*; *0.8*	**1.89 ^a^** *1.35*; *1.95*	**0.67 ^b^** *0.53*; *0.7*
**TNF-α**	**Median** *25p*; *75p*	**3.17** *2.7*; *3.3*	**4.4 ^a^** *3.7*; *7.4*	**3.23 ^b^** *2.97*; *3.55*

The effects of Ket and Ket-Tris treatment on mucosal structure in gastric tissue and changes in MPO activity [mU/(mg protein)], XOR activity [pmol/(min mg)], NO_x_ level [nmol/(mg protein)], MDA level [pmol/mL], Cyt*c* [pg/(ml protein)] and plasma TNF-α level [pg/mL]. ^a^ *p* < 0.05 between groups vs. the control group; ^b^ *p* < 0.05 between the Ket-treated group vs. the Ket-Tris-treated group.

**Table 2:** In vivo detection of the microcirculation in Study 2.

	Parameters	Control	Ket	Ket-Tris
**Stomach**	**Median** *25p*; *75p*	**882** *779*; *967*	**1084 ^a^** *982*; *1245*	**763 ^b^** *703*; *876*
**Duodenum**	**Median** *25p*; *75p*	**744** *694*; *798*	**1084 ^a^** *1015*; *1229*	**676 ^b^** *536*; *897*
**Jejunum**	**Median** *25p*; *75p*	**762** *683*; *824*	**1077 ^a^** *960*; *1267*	**754 ^b^** *679*; *805*
**Ileum**	**Median** *25p*; *75p*	**827** *742*; *906*	**1012 ^a^** *972*; *1084*	**781 ^b^** *694*; *864*
**Colon**	**Median** *25p*; *75p*	**946** *837*; *1034*	**984** *754*; *1069*	**977** *862*; *1048*

The effects of Ket and Ket-Tris treatment on changes in red blood cell velocity [µm/s]. ^a^ *p* < 0.05 between groups vs. the control group; ^b^ *p* < 0.05 between the Ket-treated group vs. the Ket-Tris-treated group.

## Data Availability

The data presented in this study are openly available in the data of the BioStudies Database data repository. This data can be found here: https://www.ebi.ac.uk/biostudies/studies/S-BSST1132 (accessed on 30 June 2023) (Title: “Conjugation with Tris decreases the risk of ketoprofen-induced mucosal damage—database”).

## Supplementary Materials

The following supporting information can be downloaded at: https://www.mdpi.com/article/10.3390/pharmaceutics15092329/s1, Figure S1: NMR spectra; File S1: ARRIVE Checklist; File S2: Wellbeing score table.
