# Supplementary material for: Conjugation with Tris Decreases the Risk of Ketoprofen-Induced Mucosal Damage and Reduces Inflammation-Associated Methane Production in a Rat Model of Colitis
Source: Pharmaceutics. 2023 Sep 16;15(9):2329. doi: 10.3390/pharmaceutics15092329 (PMC10535093; doi:10.3390/pharmaceutics15092329)

Supplementary Figure S1: NMR characterization of Ket-Tris conjugate

NMR spectrum:

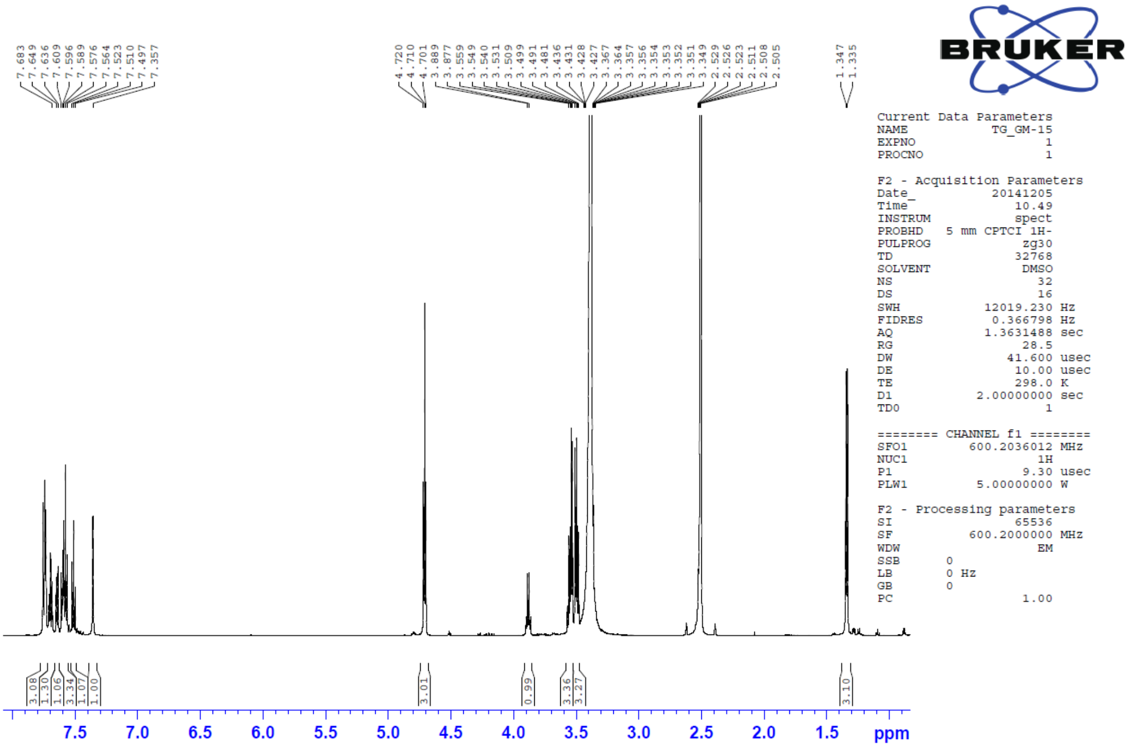

^{13}C NMR spectrum:

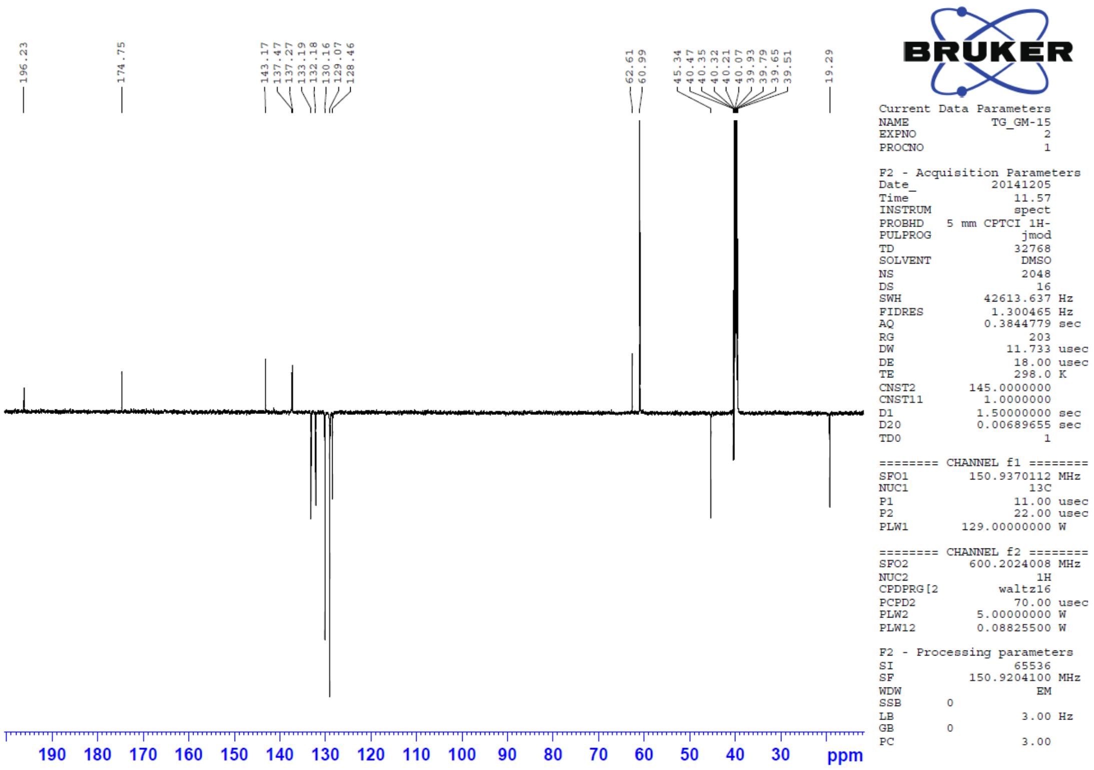

Supplement: Supplementary file 1 [file pharmaceutics-15-02329-s001.zip › Supplementary Figure S1.pdf]
