# Supplementary material for: Conjugation with Tris Decreases the Risk of Ketoprofen-Induced Mucosal Damage and Reduces Inflammation-Associated Methane Production in a Rat Model of Colitis
Source: Pharmaceutics. 2023 Sep 16;15(9):2329. doi: 10.3390/pharmaceutics15092329 (PMC10535093; doi:10.3390/pharmaceutics15092329)
Supplement: Supplementary file 1 [file pharmaceutics-15-02329-s001.zip › Supplementary File S2.pdf]

Date: From …………………. to ……………….
Experiment title:
Approval number:
Species and strain; sex:
Experiment leader:

Experimental ID:

	Score	Condition:	Day 1	Day 2	Day 3	Day 4
Condition of fur	0	not altered				
	1	piloerection, dirty fur				
Posture	0	not altered				
	1	altered weight distribution				
	2	hunched back, tremor				
Mobility/Alertness	0	not altered				
	1	slow/stiff movement, no exploratory behaviour				
	2	immobile				
Startle reflex	0	not altered				
	1	decreased, slow reaction				
	2	no reaction				
Weight	0	+ 0-5-10 gr				
	1	- 5-10 gr				
	2	- 15-20 gr				
Body temperature	0	not altered				
	1	hypothermia/fever				
Total score:						
